#  Human resource management interventions to improve health workers' performance in low and middle income countries: a realist review

**DOI:** 10.1186/1478-4505-7-7

**Published:** 2009-04-17

**Authors:** Marjolein Dieleman, Barend Gerretsen, Gert Jan van der Wilt

**Affiliations:** 1 KIT Development, Policy and Practice, Royal Tropical Institute, PO Box 95001, 1090 HA Amsterdam, the Netherlands; 2 Health Technology Assessment, Radboud University Nijmegen Medical Centre, Department of Epidemiology, Biostatistics and HTA, PO Box 9101, 6500HB Nijmegen, the Netherlands

## Abstract

**Background:**

Improving health workers' performance is vital for achieving the Millennium Development Goals. In the literature on human resource management (HRM) interventions to improve health workers' performance in Low and Middle Income Countries (LMIC), hardly any attention has been paid to the question how HRM interventions might bring about outcomes and in which contexts. Such information is, however, critical to assess the transferability of results. Our aim was to explore if realist review of published primary research provides better insight into the functioning of HRM interventions in LMIC.

**Methodology:**

A realist review not only asks whether an intervention has shown to be effective, but also through which mechanisms an intervention produces outcomes and which contextual factors appear to be of critical influence. Forty-eight published studies were reviewed.

**Results:**

The results show that HRM interventions can improve health workers' performance, but that different contexts produce different outcomes. Critical implementation aspects were involvement of local authorities, communities and management; adaptation to the local situation; and active involvement of local staff to identify and implement solutions to problems. Mechanisms that triggered change were increased knowledge and skills, feeling obliged to change and health workers' motivation. Mechanisms to contribute to motivation were health workers' awareness of local problems and staff empowerment, gaining acceptance of new information and creating a sense of belonging and respect. In addition, staff was motivated by visible improvements in quality of care and salary supplements. Only a limited variety of HRM interventions have been evaluated in the health sector in LMIC. Assumptions underlying HRM interventions are usually not made explicit, hampering our understanding of how HRM interventions work.

**Conclusion:**

Application of a realist perspective allows identifying which HRM interventions might improve performance, under which circumstances, and for which groups of health workers. To be better able to contribute to an understanding of how HRM interventions could improve health workers' performance, a combination of qualitative and quantitative research methods would be needed and the use of common indicators for evaluation and a common reporting format would be required.

## Background

The human resources crisis in the health sector in low- and middle-income countries (LMICs) is receiving increased global attention [1,2]. Policymakers and planners are realising that it is simply not possible to achieve the Millennium Development Goals if health workers' availability and performance are not addressed more effectively. Poor performance leads to inappropriate care, which contributes to reduced health outcomes, as people do not use services or are mistreated when they do. Problems relating to health workers' poor performance have been documented in various articles and reports [including [1-5]], but there is a dearth of evidence on 'what works' to improve health worker performance [4,6-8]. Moreover, evidence on effectiveness of Human Resource Management (HRM) interventions is essential, but not sufficient to assist policy makers and planners in LMIC to identify appropriate interventions to improve the performance of professional health workers in their own countries. They also need an understanding of the context within which the reported interventions produced positive outcomes as well as an insight in their mechanisms (how they worked).

Existing reviews that include HRM interventions in LMIC are limited in number and mainly identify "what works" [4,6-8], although they acknowledge the influence of the context on the outcome of interventions. Realist inquiry aims to answer the research question "what is it about this program that works for whom in what circumstances" [[9]:2]. It identifies how interventions produce certain outcomes by exploring through which mechanisms (or processes), triggered by the intervention, change is brought about, and which contextual factors are critical for success or failure. Contextual factors are the circumstances within which HRM interventions are implemented. In addition to the organizational, socio-economic, cultural and political environment, these include the stakeholders involved, their interests and convictions regarding change and the process of implementation [9]. Realist inquiry has an explanatory focus and aims to build theories about mechanisms for change. It might therefore offer a valuable addition to the current evidence-building approaches by expanding the evidence-base with information about which interventions in LMICs are successful in improving performance under which circumstances and for which groups of health workers [2,10,11].

This article systematically reviews published human resource management (HRM) interventions to improve the performance of professional health workers in LMICs, applying a realist perspective. It explores if realist review of published primary research provides better insight into the reasons why certain interventions work in certain contexts and not in others. To our knowledge we are the first to do so.

## Methodology

Based on earlier search experiences for publications on HRM interventions in LMICs which yielded very limited results, we explicitly aimed to conduct a search with high sensitivity. We searched Pubmed/medline, Ebscohost and Proquest for a 10-year period, from 1997 to October 2007, in English and French, and manually searched reference lists of relevant articles. Selection and data extraction were carried out by two researchers, independently of each other.

To be included, articles needed to report the results of an evaluation of a well-defined HRM intervention in LMIC, provide sufficient details on the research design and be published in a peer-reviewed journal. HRM interventions were defined as interventions that aim for "effective utilization of human resources in an organization" [[12]:xii]. In line with WHO (2006), we distinguished three HRM-intervention levers: job-related interventions which focus on individual occupations, support-system-related interventions, and interventions which create an enabling environment. In addition, we distinguished four dimensions of health worker performance: availability, productivity, responsiveness and competence [2]. The focus of the review was on improving performance of professional health workers, excluding interventions to increase the number of health workers through pre-service training or changes in deployment strategies and interventions targeting community health workers. We developed the search strategy based on the definition of HRM and on the dimensions of health workers' performance. We combined key words with various terms for health workers and for primary research; inclusion and exclusion criteria are listed in Appendix 1. The full search strategy can be obtained from the first author.

We systematically assessed outcome, context, and mechanisms through which the intervention produced its outcomes. Additionally, we assessed potential bias in the evaluation studies of these HRM interventions, including assessment of baseline, control group and alternative explanations for results [13]. Full details of the assessment can be obtained from the first author.

## Results

**Figure 1:**
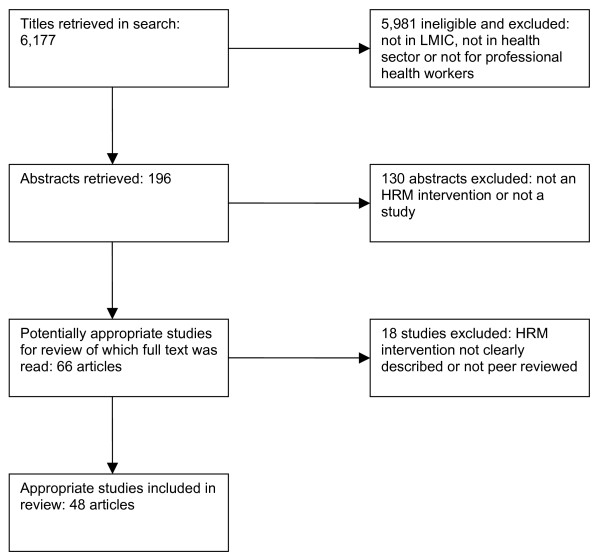
**Flowchart of search**.

We selected 48 articles for analysis from 6,177 titles (see figure 1). The interventions were categorised inductively into seven types of interventions and classified according to the three HRM-intervention levers:

- The most commonly evaluated interventions were job-related, including continuing education (n = 21) and supervision (n = 2).

- Support-system-related interventions were limited to payment of incentives (n = 4).

- Three interventions covered the creation of an enabling environment, by decentralisation of HRM functions (n = 2) and by regulations (n = 1).

- Eighteen interventions addressed all three levers, consisting of combined interventions, which included different HRM components such as training, distributing job aids and system strengthening (n = 11) and quality improvement interventions (n = 7).

Details of the included studies are provided [see Additional file 1].

### Continuing education

All 21 training courses were interactive and included field practice. The duration of the courses varied from three- to four-hour workshops (n = 4), to courses of 1–11 days (n = 16) and one distance course of 10 months.

Five studies were Randomised Controlled Trials (RCTs), eight were case control studies, and eight had a quasi-experimental design. In most cases (n = 17) results were measured by observing performance, likely to have influenced behaviour. In 12 studies, results could be partially explained by other, concurrent, interventions. Evaluation mostly (n = 13) took place within nine months of completion, making it difficult to ascertain if improved performance was sustained over time.

Overall, studies indicated that continuing education could improve knowledge, skills and performance of certain tasks in the short term. Outcome varied considerably between studies and within studies. For instance, a study in Mexico demonstrated different improvements of case management of acute respiratory infections and diarrhoea. The proportion of health care providers correctly performing specific tasks improved by 18% to 39% depending on tasks and type of provider [14]. Training in communication showed improvement in the short term [15-19]. When training included local problem solving, results could persist after nine months [19]. Continuing education of untrained (auxiliary) nurses could improve their performance [20], outperforming physicians in certain tasks [21-23].

Contextual influence was reported in various studies. Better performance after training was associated with supervision [21,22]. In India performance after training in communication declined over time likely due to poor patient flow or high administrative workload [16]. In South Africa, TB treatment outcomes only marginally improved after participatory training of nurses, because of weak management and organisational problems in facilities [24]. Integrated Management of Childhood Illness (IMCI) training was less effective in Brazil and Uganda than in Tanzania [25]. For instance, the odds ratio of a child needing antibiotics and receiving the right prescription from trained health workers as compared to untrained health workers was 4.4 in Tanzania, 2.1 in Uganda and 1.9 in Brazil [25]. Contextual factors which, according to the authors, could have contributed to differences in outcome were high staff turnover in Brazil [21,25] and abolition of user fees in Uganda [25]. In addition to health workers' training, a need to influence the context was reported in several studies, either by strengthening health systems (9×) or by developing community interventions (2×).

According to the authors, important implementation aspects of the interventions contributing to change, were:

• using a participatory approach, developing course contents based on local problems, adapting material to the local situation, and involving local stakeholders in developing and implementing the intervention (12×);

• practising tasks in the field under supervision during training, as a follow-up upon completion of training, or offering the possibility of discussing field experience after training (10×).

• developing cascade training, with health care workers trained as trainers [18,24,26].

Mechanisms through which training produced outcomes were discussed by authors in four studies and explicitly researched in three studies [15,19,24]. Improvement of health worker performance was triggered by three mechanisms: improved knowledge and skills, critical awareness on functioning of health services and being empowered to implement change. For example, Onyango-Ouma reported that training resulted in staff being more open, working better together and looking for solutions to problems, which resulted in improved provider-patient relations and reduced waiting times [19]. Lewin et al (2005) identified that training created awareness among staff to improve patient-provider relations which lead in certain instance to changes in organization of care and in others not as staff did not see themselves as agents of change [24].

### Supervision

One RCT and one case control study investigated supervision in public facilities, which was evaluated within six to eight months of completion with intrusive data collection methods. The RCT showed differences of 14% to 47% in adherence to various aspects of stock management protocols and standard treatment guidelines compared to the control groups [27]. A critical contextual factor was the presence of regular drug supplies [27]. Important implementation aspects of the intervention that contributed to change according to the authors were the use of community involvement and of participatory methods [28].

Mechanisms for change were explored by Sennun et al in Thailand [28] and discussed by Trap et al [27]. According to the authors, positive change occurred due to increased skills and knowledge. In addition, Sennun reported that change was positively influenced by health workers having a sense of belonging, as well as mutual respect between supervisors and health workers [28].

### Payment of incentives

We identified four studies that evaluated the results of paying incentives to health workers. Three of the interventions introduced user fees and paid staff from patients' fees, community cost-sharing schemes or from a drug revolving fund [29-31]. All four studies used quasi-experimental designs. Two measured long-term results (eight years and three years, respectively), and two evaluated results after one year of implementing the intervention.

The studies indicated that paying incentives can improve performance of a facility and can increase job satisfaction, staff motivation or patient satisfaction. For instance, in Cambodia, payment of staff accompanied by other interventions such as organisational changes, increased the average number of deliveries significantly from 319 to 585 per month and the average bed occupancy rate from 50.6% to 69.7% [30].

Several contextual factors were reported to influence success of the interventions. For example, utilisation of services was not necessarily influenced by user fees when patients were accustomed to paying informal fees [30], whereas utilisation of certain services dropped in urban areas in Uganda and in rural Nigeria after introduction of user fees [29,31]. In Nigeria, delay or non-payment of salaries and drug stock-outs caused a decline in staff motivation over time, with a negative influence on performance [31].

Critical aspects related to the implementation of the intervention contributing to positive outcomes, reported in these studies, include:

• availability of extra funding (3×), which can be difficult when funding depends on contributions from the community [29];

• training staff in accounting when they are responsible for financial management (1×);

• assuring results-oriented assessment linked to payments (1×); and

• support and involvement of the community in financial management (1×).

Mechanisms that lead to improved performance were researched in three studies [29-31]. The authors showed that linking individual salary supplements to functioning of health facilities can improve staff performance. The mechanism that enabled this link was staff motivation leading to development of staff initiatives to improve quality [30] or to increased presence at work [29]. In Cambodia, staff motivation to develop initiatives appeared to be a result of staff awareness that they are able to influence use and quality of care and of staff empowerment to introduce change. Self-confidence to continue developing initiatives for change was created when these changes actually improved quality of care [30]. On the contrary, in Nigeria the authors showed that staff was motivated to increase drug sales and financing due to government focus on cost recovery and health workers' interest for revenue generation; this lead to over- and irrational prescribing behaviour and a preference for curative services [31].

### Decentralisation of HRM functions

Two studies investigated the impact of decentralisation of HRM functions; one in Mozambique [32] and one in China [33]. Both studies used quasi-experimental designs. Decentralistion of HRM functions differed between the two countries; in Mozambique HRM functions were decentralised at provincial level, whereas in China hospitals were made partially responsible for HRM functions.

In China, decentralization of HRM functions lead in some hospitals to an increase in hospital income and outpatient numbers, to a reduction of hospital staff in relation to workload and employment costs and to an increase in the number of in-service training days for doctors but not for nurses [33].

The studies showed that decentralisation of HRM functions could have a positive impact on HRM, but that complementary interventions to create an enabling environment were required, such as management training, changes in bureaucratic procedures and appropriate preparation in structures and staffing [32,33]. Other examples of contextual influences were that in Mozambique the political interference of district administrators influenced transfers of health workers and administrative constraints prevented adequate performance evaluation [32]. In China, managers faced problems in addressing appropriate recruitment due to social pressure to recruit (incompetent) relatives and friends and they faced organisational pressure to increase hospital income [33].

Mechanisms that caused change in performance were partially discussed by authors of the study in China [33]. According to these authors, hospital managers in China focused on cost-recovery and on increasing hospital income. This resulted in the introduction of financial incentives that motivated doctors to over-treat and over-prescribe [33].

### Regulation

One RCT evaluated the effectiveness of inspection visits, selective punishments and the provision of regulatory documents on the practice of private pharmacies in Laos [34]. Evaluation occurred immediately after the intervention and showed improved practices, such as an increase of 34% in the availability of essential dispensing material and of 19% in order in the pharmacy. Adding intensive supervision of drug inspectors caused a significant change only in availability of essential dispensing material. Results could be partially explained by a concurrent event. Mechanisms that produced these outcomes were not discussed.

### Combined interventions

Eleven published studies on combined interventions met our criteria. These interventions all included a training component. Additional HRM components were the provision of guidelines and/or structured feedback (n = 4); feedback with enforcement of regulations or a contract (n = 3); improved monthly supervision, drug availability and guidelines (n = 2); and a comprehensive approach, with community involvement, strengthening of health systems or decentralisation of treatment at local level (n = 2).

Study designs included RCTs (n = 5), a case control study (n = 1) and quasi-experimental designs (n = 5). Nine studies evaluated within eight months of completion of the intervention, a period too short to conclude on sustained behaviour change. Nine studies had intrusive data collection methods or external, concurrent events, likely influencing results.

Results appeared to be positive in the short term. Comprehensive approaches – combining interactive and participatory training with strengthening of health systems [35-37] – showed the potential to significantly improve health workers' performance. For instance, in Bangladesh the mean index of correctly assessing sick children improved from 18 to 73 and for treatment from 8 to 54 [35]. In Morocco, the mean percentage of recommended tasks performed was 79% among the intervention group and 21% in the control group [38].

Several contextual factors were reported to influence results. For instance, in Niger, trained health workers only referred 23% of children with a general danger sign due to long distances and poor quality of referral sites [39]. In Vietnam, private pharmacies gave more weight to professional associations than in Thailand and this positively influenced their adherence to guidelines [40]. In Morocco, correct prescribing was associated with children with high fever, with younger children, with a lower patient load and with longer consultation times [38].

Critical success factors for intervention implementation were:

• including a component to strengthen health systems, such as improving drug availability, equipment and supervision; and

• involving local stakeholders such as communities, staff, local health officials or local professional associations, and adapting the intervention to the local situation (8×).

Mechanisms through which combined interventions produced positive change in health worker performance were discussed in six studies [38,40-44]. Two main mechanisms triggering change could be identified: acceptance of new information by target groups of the intervention and feeling obliged to apply new skills and knowledge in own practice. Acceptance is likely to be influenced by the perceptions on case management of professional health care providers who participated in the intervention [43], by existing clinical rules among health care providers [38] and consensus among faculty in own facility regarding clinical guidelines [42] or participation in development of guidelines [41]. Feelings among private providers that they were obliged to change was caused by establishing accountability mechanisms through social pressure and social obligation [43], through awareness raising that improved practice would improve reputation among customers [44] or through sanctions and conviction [40].

### Quality Improvement

Seven Quality Improvement (QI) interventions were identified, all using a participatory approach, analysing performance data by staff involved in service delivery, and identification and implementation of local opportunities to improve performance.

Study designs included quasi-experimental studies (n = 4), case control studies (n = 2) and one RCT, and evaluations occurred mostly (n = 4) after one year. Five research teams were either involved in the implementation of the study or used intrusive data collection methods. The results of one study might be partially attributed to a concurrent intervention.

Research indicated that QI improved the performance of tasks and case management, and that it could be successful in different contexts: QI implemented in hospitals in Ghana and Jamaica caused significant changes in obstetric care in both countries, such as an increase from 65% to 93% of patients with genital tract sepsis treated with broad-spectrum antibiotics [45].

Critical implementation aspects of the interventions contributing to success included:

• involving staff, communities and local health authorities in setting standards (3×);

• receiving support from the management of the facility and senior officials (2×); and

• using available funds and developing feasible plans for local teams (2×).

Mechanisms which triggered health workers to change were discussed in three studies [45-47]. Identified mechanisms were increased job satisfaction in El Salvador [46] and improved staff morale due to feedback meetings in Ghana and Jamaica [45] and due to community involvement and ownership in Congo [47]. In Congo additional mechanisms contributing to change were increased knowledge due to training and acceptance of indicators and willingness to adhere to self-set standards [47].

## Discussion

Our review set out to explore whether or not the application of a realist perspective to published research could improve the understanding of how HRM interventions impact on health worker performance through the analysis of the context and the mechanisms that brought about change. The findings show that HRM interventions can contribute positively to health workers' performance and the most important results were that:

• combined interventions of participatory, interactive training, job aids and strengthening health systems can be successful in improving health workers' performance;

• continuing education as a single intervention is likely to be effective in the short term and can improve the performance of untrained health care providers; however, to sustain effectiveness, additional interventions addressing health systems or community issues are required;

• QI, based on local performance analysis by teams, and payment combined with additional interventions such as organisational change, can improve health workers' performance; and

• training to identify problems and develop local solutions or to improve communication is not likely to be effective when local conditions are not addressed.

However, different contexts produced different outcomes. Commonly reported critical implementation aspects that contributed to success could be extrapolated and these were the involvement of local authorities, communities and management, adaptation to the local situation, and the active involvement of local staff to identify and implement solutions to problems. In addition, the studies provide examples of contextual factors influencing the outcome. However, it was not possible to identify patterns in how contexts influenced outcome of interventions due to their limited descriptions and the fact that there were few similar interventions implemented in different contexts.

The review teased out three mechanisms that were triggered by HRM interventions and brought about change in health workers' performance, although mechanisms were only to a limited extent discussed and even to a lesser extent researched. These mechanisms were: increased knowledge and skills, improved motivation and feeling of being obliged to change.

Increased knowledge and skills through training was an important mechanism to contribute to improved performance, but not sufficient. These findings corroborate earlier studies that continuing education is only effective to a limited extent [2,4,6-8,48,49]. The published studies reported positive outcomes when training included a participatory approach, material adapted to the local situation and practise during or after training. These intervention components indicate the use of an adult learning approach, which is reported to be effective when training adults [50]. However, only three studies explicitly reported that training was based on specific learning theories [15,19,24].

In most reported interventions, staff motivation to implement knowledge and skills appeared an additional mechanism enabling change. None of the studies reported explicitly how staff motivation was meant to be achieved or on which motivation theories HRM interventions were based. However, the studies provide some insights in mechanisms contributing to motivation. The studies show that HRM interventions triggered motivation of health care providers by:

▪ creating awareness of local problems and empowerment to develop initiatives for change [19,30] and health workers seeing themselves as agents of change [24];

▪ assuring acceptance of new information on diagnosis, treatment and care [38,41-43];

▪ creating a sense of belonging and respect [28];

▪ increasing income through financial incentives [29-31,33];

▪ providing opportunities to notice improvements in quality of care [30].

Care has to be taken to accept as a general principle that financial incentives trigger motivation which leads to improved performance. Such incentives produced negative outcomes in terms of over- prescribing and over-treating when health workers were solely rewarded by cost-recovery and revenue generation [31,33]. Non-financial rewards, such as improved patient satisfaction or patient outcomes, improved quality of care, improved relations with colleagues and managers, recognition and appreciation were only to a limited extent implemented and researched. Various studies have shown that health workers perceive non-financial incentives as more important motivators than financial incentives [among others [51-56]]. It would be interesting to evaluate the use of non-financial rewards to improve performance.

The feeling of being obliged to change was the third mechanism contributing to improved performance, mainly among private providers. This was obtained through accountability systems either by government inspection followed by sanctions [40] or by social pressure from the community resulting in improved reputation [44] and increased clientele and income [43].

The reviewed studies investigated a limited variety of HRM interventions to improve health worker performance. The most often published HRM intervention was continuing education, despite the available evidence of limited success of training as a single HRM intervention. Examples of additional HRM components that could bring about change, mainly related to staff motivation and feeling obliged to change, have been provided in this review. However, there are other HRM interventions components [2,6] which offer an opportunity to influence staff motivation, feeling obliged to change or other mechanisms that could lead to change such as job satisfaction. These interventions need to be documented, evaluated and shared. Research in high-income countries shows, that "bundles of interlinked Human Resource practices" that are aligned to the strategy and mission of an organisation are effective in enhancing workers' performance [10]. The combined and QI interventions could qualify as "bundles of HR practices", although it was not reported whether they were part of a strategic vision of the health workforce or were aligned to the strategy of the organisation or sector.

This review corroborates the findings of other reviews and demonstrates that there is evidence of positive results of HRM interventions on health worker performance, although it is limited. Other reviews acknowledge the limitations of current research and stress the need to examine the influence of the context on intervention results [4,6,7] and to consider theories of behaviour change [4]. This review showed that applying a realist perspective to the evaluation of HRM interventions offers an opportunity to deepen the understanding of HRM interventions as it includes context and mechanisms in the analysis.

Applying a realist perspective to published research has its limitations. Apart from limited reporting on the context, the process of implementation and mechanisms, the studies did not explicitly report on the underlying assumptions of how the HRM interventions should bring about change. To better understand mechanisms and to build program theories, these underlying assumptions need to be revealed and evaluated [57]. In addition, the published interventions were often evaluated at different levels (output, outcome or effect) and with different indicators, making it difficult to compare them. Because of the limited information and the missing link between evaluation and underlying assumptions of HRM interventions, these studies contribute to a limited extent to developing insights in how different HRM interventions could lead to improved health worker performance. Therefore this review is not conclusive and needs to be complemented with additional realist evaluation research so as to construct and test program theories so urgently required to assist policy makers in their choice of HRM interventions.

**Figure 2:**
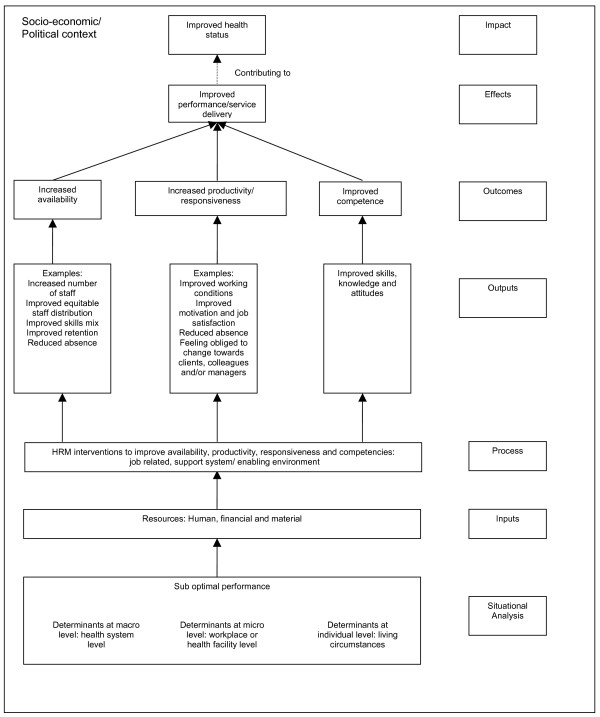
**Framework**.

We have developed a framework to facilitate understanding of mechanisms, which is based upon the dimensions of health worker performance (see figure 2, adapted from [58]). Figure 2 shows that there are a variety of interrelated mechanisms (defined in the figure as outputs) which could lead to improved availability, productivity, responsiveness and competency. In order to enable comparison of evaluation research of HRM interventions, we propose in addition the use of common indicators (see Table 1) and a common reporting format. Moreover, to gain a better understanding of outcomes, the mechanisms that caused change, and the context within which this change occurred, a combination of qualitative and quantitative research methods is needed [9].

**Table 1:** Indicators for health workers' performance

**Factors**	Examples of indicators*
*Impact*	

Health status	Decline in mortality/morbidity among targeted patients: Decreased prevalence and incidence

*Effects*	

Performance	Improved service delivery: Client satisfaction Re-admission rates and cross-infections Case fatality rates Treatment success rates, defaulter rates Coverage Service utilisation

*Outcomes*	

Availability	Waiting time, staff ratios, overtime, staff turnover, attendance of health workers

Productivity	Occupancy rate, outpatient visits and interventions provided per worker or facility Patient contacts

Competencies	Prescribing practices Adherence to protocol during diagnosis and communication with patients

Responsiveness	Proactive quality service, e.g. decubitus ulcers

*Outputs*	

Retention	Vacancies, posts filled, duration in job

Absence	Attendance of health workers, overtime

Being responsible	Showing initiative, active participation in audits and meetings Adherence to rules and Standard Operating Procedures

Skills and knowledge	Level of skills and knowledge of practices

Motivation and job satisfaction	Level of job satisfaction Level of staff motivation

Working conditions	Availability of infrastructure, medications, supplies *Being aware of and adhering to communication and decision-making procedures*: Number of meetings held with minutes and action list Confidential procedure for complaints in place and used *Management support offered*: Amount of supportive supervision

* Indicators taken from among others:

• WHO: *World health report 2006*. *Working together for health*. Geneva, World Health Organization; 2006

• Buchan J.: *Increasing the productivity of an existing "stock" of health workers: review for DFID*. London, Department for International Development; 2005.

• Hornby P, Forte P.: *Guidelines for introducing human resource indicators to monitor health service performance*. Keele, Keele University Centre for Health Planning and Management; 2002.

Adapted from Dieleman M. and Harnmeijer JW.: *Improving health worker performance, in search of promising practices*. Amsterdam, The Netherlands: KIT; 2006

This review is restricted to articles in English and French and might have missed publications in other languages. In addition, we do not know to what extent our review suffers from publication bias. To overcome this problem in the future, registration of protocols should be considered, as is currently the case for clinical trials.

## Conclusion

Applying a realist perspective to the review of published HRM interventions offers an opportunity to gain a better understanding of how different HRM interventions can improve performance, under which circumstances and for which groups of health workers. To improve health workers' performance, health managers need insight into the context within which interventions achieved results elsewhere and an understanding of the mechanisms that triggered change. This review showed that the current evidence-base insufficiently contributes to the development of these insights and that the application of a realist perspective to HRM evaluations and reviews could be a valuable addition to the existing evaluation methods.

## Competing interests

The authors declare that they have no competing interests.

## Authors' contributions

MD designed the study, participated in the selection of articles and the analysis of the data, and drafted the manuscript. BG substantially commented on the draft manuscripts. GJvdW advised on the study design, participated in the selection of articles and the analysis of the data, and substantially commented on draft versions of the manuscript. All authors read and approved the final manuscript.

## Appendix 1 search strategy and inclusion and exclusion criteria

### Search strategy

#### Use of key words

1. Key words, related to HRM interventions to improve health workers' performance in terms of productivity, responsiveness and competence:

Personnel management, performance management, supervision, recognition, professional development, continuing education, training, quality assurance, quality improvement, performance improvement, performance appraisal, incentives, allowances, guidelines, tools, support, reward, sanctions, leadership, participation, integration of services, remuneration, payment, performance-based incentives, equipment, technologies, supplies, workflow, workload, workplace safety, medical care, integration of services, decentralisation, teamwork, contract, performance contract.

2. Key words related to health workers:

Health care providers, health workers, health service providers, nurses, doctors, pharmacists, private practitioners, public-sector health care providers, private health care providers.

3. Key words related to primary research:

Randomised controlled trials, qualitative studies, intervention studies, evaluation.

The full search strategy can be obtained from the first author.

### Inclusion criteria and exclusion criteria

#### Inclusion criteria

Health workers are limited to professionally trained health cadres, such as medical doctors, nurses, laboratory technicians, midwives etc.

#### Exclusion criteria

Not included are articles on HRM interventions to improve performance of lay health workers or to increase availability of professional health workers, in more detail:

▪ graduate training programmes where people leave their workplace for a year or more

▪ students preparing for health professions

▪ development of a training programme

▪ programmes for volunteers or for community health workers

▪ interventions to improve the skills mix in the workplace

▪ interventions to improve attraction and retention, with the limitation that some HRM practices to enhance job satisfaction and motivation are likely to have an impact on retention and vice versa – articles reporting on effects related to increased performance have been included

▪ interventions to improve recruitment of professional health workers

▪ interventions to test job aids such as guidelines, treatment protocols etc (as opposed to the use of job aids to improve performance)

## Supplementary Material

Additional file 1 **Overview of studies included in this review**. This table provides an overview of the studies included in the review. Click here for file
