# Supplementary material for: Human resource management interventions to improve health workers' performance in low and middle income countries: a realist review
Source: Health Res Policy Syst. 2009 Apr 17;7:7. doi: 10.1186/1478-4505-7-7 (PMC2672945; doi:10.1186/1478-4505-7-7)
Supplement: Additional file 1 — Overview of studies included in this review. This table provides an overview of the studies included in the review. [file 1478-4505-7-7-S1.doc]

| **Type of HRM intervention** | **No. of studies** | **Topics covered** | **Authors and year of publication** | Intervention levers* | | | **Explicit theoretical underpinning: using models or international literature** |
| --- | --- | --- | --- | --- | --- | --- | --- |
|  |  |  |  | Job-related | Support systems | Enabling |  |
| Continuous education/  in-service training | 21 | 6 General health care | Roter et al, 1998; Diprete Brown et al, 2000; Meyer et al, 2001; Onyango-Ouma et al, 2001a; Onyango-Ouma et al, 2001b; Agyepong et al, 2002 | x |  |  | 4 Behavioural models  8 Embedded in international literature on training effectiveness  9 Nothing stated |
|  |  | 8 IMCI | Simoes, 1997; Amaral et al, 2004; Gouws, 2004; Tanzania IMCI multi-country evaluation health facility survey study group, 2004; Mohan et al, 2004; Amaral et al, 2005; Gilroy et al, 2004; Pariyo et al, 2005 |  |  |  |  |
|  |  | 2 Specific areas of child care | Bojalil et al, 1999; Flores, 2002 |  |  |  |  |
|  |  | 1 TB control | Lewin et al, 2005 |  |  |  |  |
|  |  | 2 Other disease control | Mahé, 2005; Mock, 2005 |  |  |  |  |
|  |  | 2 STD/HIV/AIDS | Garcia et al, 1998; Ezedinachi et al, 2002 |  |  |  |  |
| Supervision | 2 | 2 General health care | Trap et al, 2001, Sennun et al, 2006 | x |  |  | 2 Embedded in international literature on community participation |
| Payment of incentives | 4 | 3 General health care | Kipp et al, 2001; Akashi et al, 2004; Uzochukwe et al, 2005 |  | x |  | 2 Embedded in international literature on effects of payment on performance  2 Nothing stated |
|  |  | 1 TB Control | Harries et al, 2005 |  |  |  |  |
| Decentralisation | 2 | 2 General health care | Saide et al, 2001; Liu et al, 2006 |  |  | x | 2 Embedded in international literature on decentralisation and HRM |
| Regulations | 1 | 1 General health care | Stenson et al, 2001 |  |  | x | 1 Embedded in international literature on regulations |
| Combined approaches | 11 | 4 General health care | Thamlikitkul et al, 1998; Diallo et al, 2002; Chalker et al, 2002; Chalker et al, 2004 | x | x | x | 6 Embedded in international literature on success of interventions  5 Nothing stated |
|  |  | 4 IMCI | Tawfik et al, 2001; Kelley et al, 2001; El Arifeen, 2004; Naimoli et al, 2006 |  |  |  |  |
|  |  | 1 Specific area of child care | Chakraborty et al, 2000 |  |  |  |  |
|  |  | 1 TB control | Thiam et al, 2007 |  |  |  |  |
|  |  | 1 STD | Harrison et al, 2000 |  |  |  |  |
| Quality Assurance/ Quality Improvement | 7 | 2 General health care | Wahlstrom et al, 2003; du Mortier and Arpagus, 2005 | x | x | x | 6 Embedded in international literature on QA, tools and frameworks  1 Nothing stated |
|  |  | 1 Specific area of child care | Kelley et al, 2003 |  |  |  |  |
|  |  | 4 SRH/  maternal care | Wilkinson, 1997; Wagaarachchi et al, 2001; Hermida and Robalino, 2002; Agurto et al, 2006 |  |  |  |  |

* According to WHO: p.71–86 [2]:

- Job-related interventions which focus on individual occupations; examples are job descriptions, supervision and training;
- Support-system-related interventions; examples are remuneration, infrastructure, supplies and communication;
- Interventions which create an enabling environment and focus on managerial culture and organisational arrangements; examples are team management, responsibility and accountability.
